# Potential Therapies for Cerebral Edema After Ischemic Stroke: A Mini Review

**DOI:** 10.3389/fnagi.2020.618819

**Published:** 2021-02-04

**Authors:** Yi Yao, Yonggang Zhang, Xiaoyang Liao, Rong Yang, Yi Lei, Jianzhao Luo

**Affiliations:** ^1^International Medical Center, Ward of General Practice and National Clinical Research Center for Geriatrics, West China Hospital, Sichuan University, Chengdu, China; ^2^Department of Periodical Press and National Clinical Research Center for Geriatrics, West China Hospital, Sichuan University, Chengdu, China; ^3^Nursing Key Laboratory of Sichuan Province, Chengdu, China; ^4^Chinese Evidence-Based Medicine Center, West China Hospital, Sichuan University, Chengdu, China

**Keywords:** ischemia stroke, cerebral edema, molecular mechanism, drug therapy, preclinical drug evaluation, clinical trial

## Abstract

Stroke is the leading cause of global mortality and disability. Cerebral edema and intracranial hypertension are common complications of cerebral infarction and the major causes of mortality. The formation of cerebral edema includes three stages (cytotoxic edema, ionic edema, and vasogenic edema), which involve multiple proteins and ion channels. A range of therapeutic agents that successfully target cerebral edema have been developed in animal studies, some of which have been assessed in clinical trials. Herein, we review the mechanisms of cerebral edema and the research progress of anti-edema therapies for use after ischemic stroke.

## Introduction

Stroke is the leading cause of death worldwide (Feigin et al., 2015). Ischemic stroke accounts for 69.6–70.8% of all strokes (Wang W. et al., 2017). The mortality rate of patients with acute ischemic stroke is as high as 2.3–3.2% within 1 month after onset (Huang et al., 2010; Wang D. et al., 2017; Li et al., 2019; He et al., 2020). The formation of cerebral edema is a major cause of death in stroke patients (Simard et al., 2007; Walcott et al., 2014; Rungta et al., 2015).

Cerebral edema is extremely dangerous. Severe cerebral edema after stroke can increase the mortality rate to 80% and is an important predictor of poor prognosis (Kochanek et al., 2011; Battey et al., 2014; Walcott et al., 2014; Nawabi et al., 2019). Thus, an effective treatment of cerebral edema during the early phase of stroke is particularly important. The human brain is enclosed in a hard skull and the interaction between swollen brain tissue and the skull after an ischemic stroke can further increase intracranial pressure. This increase in intracranial pressure can also increase the degree of cerebral hypoxia and ischemia. Unfortunately, the current osmotic therapies focus on treating edema rather than preventing it, and evidence for efficacy remains insufficient (Torbey et al., 2015). Early craniotomy decompression reduces mortality and improves prognosis (Shah et al., 2019), but it deals with downstream events rather than targeting the underlying molecular mechanisms of cerebral edema (Stokum et al., 2015).

Experimentally, a number of agents have been developed that successfully target cerebral edema, one of which has entered clinical trials. This article reviews the mechanisms of cerebral edema and the research progress of anti-edema drugs.

## Mechanisms of Cerebral Edema

Cerebral edema after an ischemic stroke includes cytotoxic edema, ionic edema, and vasogenic edema (Liebeskind et al., 2019). These three processes are closely linked and, eventually, ~8.5–30% of patients develop cerebral hemorrhage (Lindley et al., 2004; Paciaroni et al., 2008). However, the mechanisms of cerebral edema formation are not fully understood.

**Figure 1:**
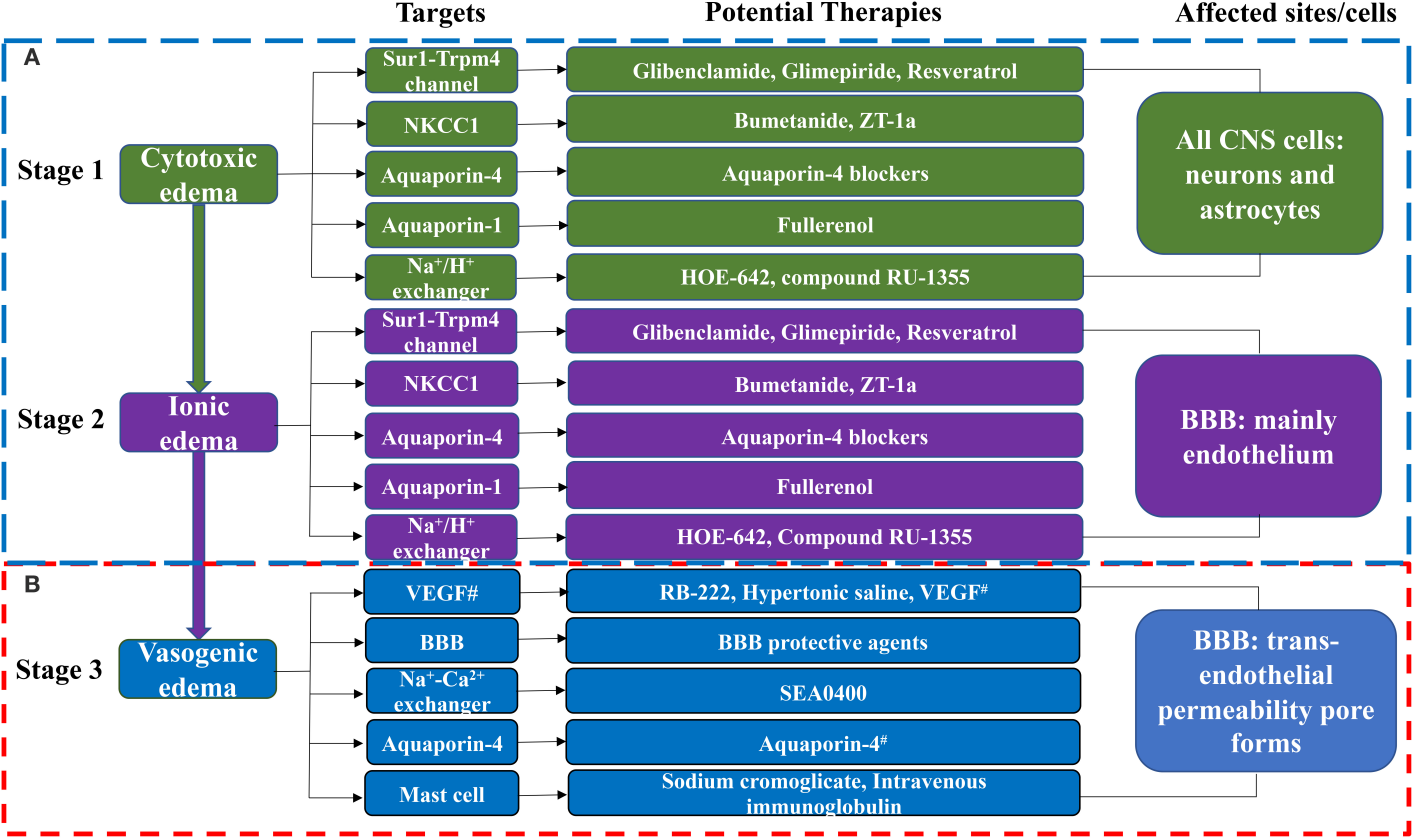
The molecular targets and anti-edema therapies in ischemic stroke. **(A)** The blood-brain barrier remains intact in this phase. **(B)** The blood-brain barrier is destroyed in this phase. CNS, Central Nervous System. BBB, blood-brain barrier; NKCC1, Na^+^-K^+^-2Cl^−^ cotransporter-1; ZT-1a, a highly effective and selective SPAK inhibitor; HOE-642, a Na^+^-H^+^ exchanger inhibitor; Compound RU-1355, a Na^+^-H^+^ exchanger inhibitor; RB-222, anti-VEGF neutralizing antibody; SEA0400, a specific inhibitor of the Na^+^-Ca^2+^ exchanger; T3, thyroid hormone; VEGF, vascular endothelial growth factor; Anti-miR-1, anti-microRNA-1; Antagomir, inhibitor of miRNA-1. ^#^Note that VEGF can both promote brain edema and have neuroprotective effects. Aquaporin-4 has a protective effect against vasogenic edema. Aquaporin-4 blockers include T3, Inhaled H2S, Acetazolamide, Lactosides B, Mitochondrial aldehyde dehydrogenase 2, Paeoniflorin, Astragaloside IV, and Tongxinluo. BBB protective agents include Alpha-tocopherol, 8-methoxypsoralen, 3-aminobenzamide, Anti-miR-1 or MicroRNA-1 antagomir, MicroRNA-132, MicroRNA-1906, chemokine-like factors, magnesium sulfate, ulinastatin, ginkgo diterpene lactone meglumine injection, olaparib, recombinant human erythropoietin, and 1-trifuoromethoxyphenyl-3-(1-propionylpiperidin-4-yl) urea.

The formation of cerebral edema is still based on the Starling principle. Cytotoxic edema refers to the brain swelling caused by ions (Na^+^, Cl^−^) and water entering the cells of neurons or astrocytes (Rungta et al., 2015). Cytotoxic edema occurs quickly after brain tissue ischemia, causing intracellular swelling without increasing the brain tissue volume (Liebeskind et al., 2019). Therefore, cytotoxic edema is intracellular edema. Astrocytes in the brain are most affected by cytotoxic edema (Stokum et al., 2016). Cytotoxic edema leads to changes in ion concentrations on both sides of the blood–brain barrier (BBB), with the intravascular Na^+^ concentration increasing above that in the interstitium (Mori et al., 2002). The new ionic gradients provide the driving force for ionized edema and vasogenic edema. The sulfonylurea receptor 1-immediate receptor potential melatonin 4 (Sur1-Trpm4), the Na^+^-K^+^-2Cl^−^ cotransporter protein-1 (NKCC1), aquaporin-4 (AQP4), the Na^+^-H^+^ exchanger, and the Na^+^-Ca^2+^ exchanger drive this process (Douglas et al., 2001; Amiry-Moghaddam et al., 2004; Hamann et al., 2010; Xue and Haddad, 2010; Ferrazzano et al., 2011; Jayakumar et al., 2014; Stokum et al., 2016) (Figure 1).

Subsequently, cerebral edema enters the stage of ionic edema. Ionic edema is caused by water and ions crossing the BBB from the vasculature into the brain (Simard et al., 2007). Ionic edema is a subtype of extracellular edema lacking albumin and has been defined in recent years independently of cytotoxic edema. The BBB remains intact in this stage, and ionic edema is completely driven by various ion channels and transporters in the BBB, including the Sur1-Trpm4 channel, NKCC1, Na^+^-K^+^-cotransporter (KCC), the Na^+^-H^+^ exchanger, and AQP4 (Nilius and Droogmans, 2001; Dolman et al., 2005; Yang et al., 2008; Lam et al., 2009; Previch et al., 2016) (Figure 1).

Following the destruction of BBB, physical connections are made between blood vessels and the interstitium of the brain. Plasma proteins and other macromolecules pass through the BBB into the extracellular space of the brain (Stokum et al., 2016). Vasogenic edema may be considered to be an ultrafiltrate of blood. Vascular endothelial growth factor (VEGF) and free radical metalloprotease (MMP) are involved in vasogenic edema (Zhang et al., 2000; Yang et al., 2007). Mast cells are also involved in this process (Parrella et al., 2019). By contrast, AQP4 helps to clear vasogenic edema (Papadopoulos et al., 2004) (Figure 1).

## Sur1-Trpm4 Channel Inhibitors

The Sur1-Trpm4 channel is widely involved in both cytotoxic and ionogenic edema and is the most promising therapeutic target for edema treatment. The Sur1-Trpm4 channel inhibitors are the only drugs to enter clinical trials for the treatment of cerebral edema after ischemic stroke (Table 1).

**Table 1:** Basic information and clinical characteristics of clinical trials of glimepiride.

**Identifier**	**Year**	**Phase**	**Subject information**	**Primary outcome**	**Status**	**Intervention**	**Conclusion**
NCT01132703	2010	I	34 health human volunteers	Adverse events. Hypoglycemia and EKG changes	Completed	RP-1127 Placebo	Two patients had persistent hypoglycemia. No serious adverse events.
NCT01268683 GAMES Pilot	2012	II	10 participants, clinical diagnosis of anterior circulation of ischemic stroke, baseline MRI DWI volume of 82–210 cm^3^, 18–70 years of age, and the start of drug infusion ≤ 10 h from symptom onset.	Rate of recruitment. Safety and tolerability. Pharmacokinetics/Pharmacodynamics. Clinical and MRI outcome data	Completed	RP-1127	Reduce the cerebral edema, the drug is well-tolerated, without safety risks and severe hypoglycemia side effects.
NCT01794182 GAMES-RP	2015 (Estimated)		83 patients, clinical diagnosis of acute severe anterior circulation ischemic stroke, baseline DWI lesion volume of 82–300 cm^3^, age 18–80 years, and time of symptom onset to start of drug infusion of ≤ 10 h.	The proportion of patients with a mRS at day 90 ≤4 without decompressive craniectomy. Safety of the drug	uncompleted. end early for financial reasons	RP-1127 Placebo	Reduce cerebral edema, midline shift and plasma MMP-9 concentration, does not significantly reduce mortality
NCT02864953	2021 (Estimated)	III	Estimated 680 participants. Patients with large (MRI-DWI 80–300 cm^3^) acute MCA ischemic stroke or large hemispheric infarction with NIHSS ≥10, study treatment infusion within 10 h after time of symptom onset. Participants receive thrombectomy, which must be based on post-thrombectomy MRI-DWI	The proportion of participants with improvement in functional outcome at day 90 assessed *via* the mRS	Recruiting	BIIB093 Placebo	No conclusion

*RP-1127, glyburide for injection; MRI, magnetic resonance imaging; DWI, diffusion-weighted imaging; BIIB093, intravenous glyburide; NIHSS, National Institutes of Health stroke scale; MCA, middle cerebral artery; MMP-9, matrix metallopeptidase 9; mRS, modified Rankin Scale; EKG, electrocardiogram; IV, Intravenous injection; rtPA, recombinant-tissue plasminogen activator; GAMES Pilot, Glyburide Advantage in Malignant Edema and Stroke-Pilot; GAMES-RP, Glyburide Advantage in Malignant Edema and Stroke—Remedy Pharmaceuticals*.

### Glibenclamide

In a phase I trial of intravenous glibenclamide in normal volunteers, two of the 34 subjects in the high dose group had persistent hypoglycemia, while none reported serious adverse events. In a phase II trial of intravenous glibenclamide in 10 patients, there was a reduction in cerebral edema (Sheth et al., 2014a,b), and glibenclamide was well tolerated with no severe hypoglycemia. The phase II trial of intravenous glibenclamide (Sheth et al., 2016) also reported reduced cerebral edema with a lower midline shift but showed no reduction in mortality. Post-exploratory analyses of that trial suggested that glibenclamide could reduce water accumulation, the mass effect, improve survival, decrease midline deviation, and reduce MMP-9 expression (Kimberly et al., 2018; Sheth et al., 2018; Vorasayan et al., 2019). A phase III trial examining whether intravenous glibenclamide can improve functional outcomes at 90 days is currently underway (Table 1).

Glibenclamide requires intravenous administration, which limits its application in the out-of-hospital setting. However, oral preparations can be administered in the early stages of edema. For example, in a study exploring the relationship between oral glibenclamide and cerebral edema in 213 patients with ischemic stroke and 40 patients in a matched cohort (Huang et al., 2019), oral glibenclamide did not increase early death or hypoglycemia but prevented cerebral edema. However, that study did not examine the optimal drug dose or compare the efficacy of oral vs. intravenous glibenclamide administration.

### Glibenclamide Combined With Other Therapies

At present, there are no anti-edema drugs approved for clinical practice. The BBB can limit drug entry into the brain, resulting in low central drug concentrations (Patel et al., 2012). Furthermore, cerebral edema is caused by multiple factors and mechanisms. As such, a single target drug may not effectively prevent the formation of cerebral edema (Deng et al., 2019). Deng et al. (2019) reported that nanoparticles of betulinic acid from the Chinese herb *Eucommia ulmoides* could be loaded with glibenclamide and could effectively penetrate the BBB to reduce infarct size and cerebral edema in mice. Furthermore, this system allowed a lower effective dose of glibenclamide, targeted to Sur1-Trpm4 and oxidation, and improved the anti-cerebral edema efficacy of glibenclamide. In another study, the combined treatment with glibenclamide and therapeutic hypothermia showed a synergistic neuroprotective effect by reducing edema and improving neurobehavioral function in a middle cerebral artery occlusion (MCAO) model in rats and in an oxygen and glucose deprivation-reoxygenation model in endothelial cells (Zhu et al., 2018).

### Glimepiride

Preclinical and clinical studies have shown that glibenclamide can reduce cerebral edema but can cause severe hypoglycemia. However, glimepiride, the latest second-generation sulfonylurea, has reduced hypoglycemic actions (Holstein et al., 2001). Glimepiride treatment can reduce stroke in mice (Darsalia et al., 2013) and be as effective as glibenclamide in reducing cerebral edema in wild-type mice (Wang X. et al., 2020). However, in that study, glimepiride was administered at 40 min before reperfusion, which is not feasible in clinical practice. Thus, further studies are required to assess its clinical potential.

### Sur1-Trpm4 Inhibitors

A number of drugs that block the Sur1-Trpm4 channel have been developed. For example, resveratrol is a natural product found in a range of plants. Resveratrol has antioxidant actions and can protect against cerebral ischemia, reduce cerebral edema, and prevent BBB damage (Ataie et al., 2016; Pineda-Ramírez et al., 2018, 2020). Furthermore, resveratrol was reported to reduce the expression of Sur1 and AQP4 and the formation of edema (Alquisiras-Burgos et al., 2020).

## Vascular Endothelial Growth Factor-Related Drugs

Vascular endothelial growth factor is a mitogen that promotes the formation of new blood vessels, protects nerves, and increases capillary permeability. VEGF when used before the model was reported to reduce the infarct volume and cerebral edema in a rat MCAO model (Harrigan et al., 2003), despite enhancing capillary permeability. The combination therapy of VEGF and nerve growth factor could also reduce infarct volume and cerebral edema, with better efficacy with earlier administration in rabbits (Yang et al., 2018). By contrast, VEGF treatment after cerebral ischemia was found to increase BBB permeability and leakage and aggravate cerebral edema (Chi et al., 2005). Furthermore, Kim et al. (2018) reported that VEGF could promote the formation of cerebral edema in patients with stroke. These contrasting findings may be related to differences in the timing of VEGF administration between the studies.

Interestingly, intraventricular injection of an anti-VEGF neutralizing antibody (RB-222) reduced the number of immature blood vessels following cerebral edema in a rat MCAO model (Zhang et al., 2017). Furthermore, hypertonic saline could reduce osmotic pressure and reduce the formation of cerebral edema *via* downregulating NKCC expression and inhibiting the VEGF (Huang et al., 2014). Finally, a treatment with 10% hypertonic saline was associated with the downregulation of zonula occludens 1 and occludin expression *via* the inhibition of the VEGF receptor 2/phospholipase cγ1/endothelial nitric oxide synthase pathway (Wang et al., 2019).

## Aquaporin Blockers

Aquaporins are known to contribute to cytotoxic edema. In particular, AQP4 is widely involved in water balance in patients with stroke (Vella et al., 2015; Verkman et al., 2017), with increased expression at 6 h after cerebral infarction and a peak at 3 days (Wei et al., 2015). AQP4 has bidirectional effects of water transport, which is involved in both the formation and the removal of cerebral edema (Stokum et al., 2015). AQP4 promotes the formation of cerebral edema in the early stage (cytotoxic and ionic edema; Tang and Yang, 2016). The brain edema of AQP4-deficient mice was decreased by 35% compared to the wild-type mice (Manley et al., 2000). However, another study had found that AQP4 exacerbates the formation of cerebral edema (Zeng et al., 2012). When cerebral edema enters the stage of vasogenic edema, water enters through the incomplete space between vascular endothelial cells. Cerebral edema is no longer closely related to AQP4, which is more involved in the clearance of cerebral edema (Tang and Yang, 2016). Unfortunately, the boundary between cytotoxic edema, ionic edema, and vasogenic edema is not obvious. However, how to determine the starting and ending time of APQ4 inhibitor is a problem to be solved in the later research. Treatment with thyroid hormone also reduced cerebral edema by inhibiting AQP4 (Mdzinarishvili et al., 2013; Sadana et al., 2015) and may be neuroprotective in patients with stroke. Experimentally, Wei et al. (2015) reported that inhaled hydrogen sulfide (H2S) could reduce cerebral edema in rats by inhibiting AQP4, as well as protect the BBB. In a permanent-MCAO model, the combined treatment with aquaporin inhibitors and cerebrolysin also reduced the formation of cerebral edema (Catalin et al., 2018). Furthermore, acetazolamide (Duan et al., 2019), lactosides B, mitochondrial aldehyde dehydrogenase 2 (Li et al., 2018), paeoniflorin, astragaloside IV (Chu et al., 2017), and the Chinese herbs show the neuroprotective effect (Ni et al., 2020), while Tongxinluo (Cai et al., 2016) showed anti-edema actions *via* the inhibition of AQP4.

Aquaporin-1(AQP1) is also involved in the formation of cerebral edema (Qiu et al., 2014), while AQP1 inhibitors, such as fullerenols, can reduce cerebral edema (Darabi and Mohammadi, 2017). However, AQP1 is expressed in different species (Arciénega et al., 2010; Badaut et al., 2011) and different organs (Buffoli, 2010), which limits the use of AQP1 inhibitors. Importantly, while aquaporins are involved in cytotoxic edema, the inhibition of AQPs is only effective in the early stage of the disease.

## ION Channel Blockers

### Cation-Cl^−^ Cotransporter Inhibitors

Na^+^-K^+^-2Cl^−^ cotransporter protein-1 and KCC2 play important roles in the formation of both cytotoxic and ionic edema. NKCC1 and KCC2 are cationic Cl^−^ cotransporters that have opposing actions on intracranial water and electrolyte balance (Russell, 2000; Blaesse et al., 2009). Bumetanide, which inhibits NKCC1, can reduce cytotoxic edema (Yan et al., 2003). Furthermore, Wang et al. (2014) reported that bumetanide reduced cerebral edema in an ischemia-reperfusion model without changing the KCC2 protein expression. Bumetanide plays a role in both the acute and chronic phases of cerebral ischemia (Xu et al., 2016, 2017). Zhang et al. (2020a) also found that ZT-1a, a novel selective SPS1-related proline/alanine-rich kinase (SPAK) inhibitor that inhibits NKCC1 while activating KCC2, reduced cerebral edema and improved stroke prognosis.

### Na^+^-H^+^ Exchanger Inhibitors

As for NKCC1, the Na^+^-H^+^ exchanger plays an important role in the early stage of cerebral edema after ischemia. Hypoxia can stimulate BBB Na^+^-H^+^ exchanger activity, thereby promoting the formation of cerebral edema. Intravenous injection of a Na^+^-H^+^ exchanger inhibitor (HOE-642; compound RU-1355) significantly reduced the Na^+^ concentration and reduced cerebral edema (O'Donnell et al., 2013; Spasov et al., 2016). Interestingly, a combined treatment with HOE-642 and bumetanide had similar efficacy to HOE-642 alone, suggesting no additive effect of two types of drugs.

### Na^+^-Ca^2+^ Exchanger Inhibitors

Maintaining intracellular Ca^2+^ homeostasis is important for protecting the BBB. Koyama et al. (2004) found that the Na^+^-Ca^2+^ exchanger was involved in vasogenic edema and BBB destruction. Furthermore, Na^+^-Ca^2+^ exchanger inhibitors (SEA0400) could reduce the formation of cerebral edema and cerebral infarction volume after cerebral ischemia (Matsuda et al., 2001).

## Conivaptan

Arginine vasopressin and its receptors, V1a and V2, play important roles in cerebral edema after ischemic stroke (Vakili et al., 2005). Conivaptan is a V1a and V2 receptor blocker that can reduce cerebral edema (Ansari et al., 2018). Intraperitoneal administration of conivaptan was reported to be superior to continuous intravenous administration (Zeynalov et al., 2016). Zeynalov et al. (2017) also found that conivaptan treatment at 3 h after ischemia could reduce edema, suggesting a role in early cytotoxic edema.

## MicroRNAs

MicroRNAs (miRNAs) regulate the gene expression at the transcriptional level and are promising targets for disease therapy (Carleton et al., 2007; Li G. et al., 2018). The expression of miRNA-1 is related to ischemic damage and cellular apoptosis (Chen et al., 2006). Treatment with anti-miR-1 reduced the infarct volume (Selvamani et al., 2012), while treatment with the miRNA-1 antagomir significantly reduced cerebral edema and BBB damage (Talebi et al., 2019). Of note, other miRNAs, including miRNA-132 (Zuo et al., 2019) and miRNA-1906 (Yu and Li, 2020), are potential targets for the treatment of edema.

## BBB Protective Agents

Vasogenic edema is accompanied by BBB destruction. Thus, maintaining BBB integrity is an important outcome measure for anti-edema treatments. Alpha-tocopherol can protect the BBB *via* its antioxidant actions (Chaudhary et al., 2003; Hsiao et al., 2007) and reduce edema formation after ischemic stroke (Azar et al., 2017). Treatment with 8-methoxypsoralen can also improve BBB ultrastructure by increasing the NFE2-related factor 2 and hemeoxygenase 1 protein expression, thus reducing edema (Liu et al., 2018). Furthermore, 3-aminobenzamide and calcitriol can both maintain BBB integrity and have anti-edema effects (Sadeghian et al., 2019; Wang J. et al., 2020). Mast cells are also involved in BBB destruction (Ocak et al., 2019), while the modulation of mast cells with agents such as sodium cromoglycate (McKittrick et al., 2015) and intravenous immunoglobulin (Widiapradja et al., 2012) can reduce edema. Furthermore, chemokine-like factors can maintain the BBB integrity and inhibit the formation of cerebral edema (Kong et al., 2016). Potential drugs targeting BBB include magnesium sulfate (Shadman et al., 2019), ulinastatin (Li X.-F. et al., 2018), ginkgo diterpene lactone meglumine injection (Li et al., 2017), olaparib (Teng et al., 2016), recombinant human erythropoietin (Wang et al., 2015), and 1-trifuoromethoxyphenyl-3-(1-propionylpiperidin-4-yl) urea (Zhang et al., 2020b).

## Perspectives

Cerebral edema following ischemic stroke is associated with a poor prognosis. Unfortunately, there are currently limited specific anti-cerebral edema treatment options available for clinical use. Traditional osmotic therapies are not specific to the molecular mechanism of cerebral edema. Their main role is to reduce intracranial hypertension and relieve mass effects (Stokum et al., 2020), which may cause serious complications such as water and electrolyte disorders and kidney damage (Zhang et al., 2019). These drugs are usually used only when high levels of intracranial hypertension are reached and cerebral perfusion is threatened. Potential new drugs target the pathophysiological mechanism of cerebral edema formation and prevent the formation of cerebral edema while avoiding the above complications.

A range of therapeutic agents (Table 2) have been developed that successfully target cerebral edema and reduce brain injury in animal models. These include targeted ion channels, transporters, and specific targets. Also include anti-apoptotic, anti-inflammatory, and antioxidant drugs (Tan et al., 2019; Moghadam and Fereidoni, 2020; Yang et al., 2020). The Sur1-Trpm4 inhibitors are of particular interest and have been studied in three clinical trials. However, as of yet, no drugs have been approved, which may relate to the design of the preclinical animal experiments. To improve the clinical translation, further preclinical animal studies are required in a range of models, which should examine the optimal therapeutic windows for the various agents. It is also important to consider that cerebral edema is caused by multiple mechanisms and that combination therapies may be the most effective treatment strategy.

**Table 2:** The mechanisms of cerebral edema and the various anti-edema agents.

**Type of cerebral edema**	**The affected site and features**	**Ion channel, transporters, or target**	**Agents**
Cytotoxic edema	All CNS cell types. Especially the astrocytes. Ions and water flow into the cells. The cells swelled. An ion concentration gradient is formed between the capillaries and the brain parenchyma.	Sur1-Trpm4 channel	Oral glibenclamide, Intravenous glibenclamide (RP-1127, BIIB093), Glimepiride, Resveratrol
		NKCC1	Bumetanide, ZT-1a
		Aquaporin-4	Thyroid hormone (T3), Inhaled H2S, Acetazolamide, Lactosides B, Mitochondrial aldehyde dehydrogenase 2, Paeoniflorin and astragaloside IV, Tongxinluo
		Aquaporin-1	Fullerenol
		Na^+^/H^+^ exchanger	HOE-642, Compound RU-1355
Ionic edema	Endothelial cells. Ions and water flow into the brain parenchyma from the blood vessels. In this process, the blood-brain barrier remains intact, and a variety of ion channels and transport proteins participate in it.	Sur1-Trpm4 channel	Oral glibenclamide, Intravenous glibenclamide (RP-1127, BIIB093), Glimepiride, Resveratrol
		NKCC1	Bumetanide, ZT-1a
		Na^+^/H^+^ exchanger	HOE-642, Compound RU-1355
		Aquaporin-4	Thyroid hormone (T3), Inhaled H2S, Acetazolamide, Lactosides B, Mitochondrial aldehyde dehydrogenase 2, Paeoniflorin and astragaloside IV, Tongxinluo
		Aquaporin-1	Fullerenol
Vasogenic edema	BBB.	VEGF^#^	RB-222, Hypertonic saline, VEGF^#^
	The integrity of the blood-brain barrier is destroyed, and plasma proteins and water penetrate into the brain tissue	BBB	Alpha-tocopherol, 8-methoxypsoralen, 3-aminobenzamide, Anti-miR-1 or MicroRNA-1 antagomir, MicroRNA-132, MicroRNA-1906, Chemokine-like factors, Magnesium sulfate, Ulinastatin, Ginkgo diterpene lactone meglumine injection, Olaparib, Recombinant human erythropoietin, 1-trifuoromethoxyphenyl-3-(1-propionylpiperidin-4-yl) urea.
		Na^+^-Ca^2+^ exchanger	SEA0400
		Aquaporin-4	Aquaporin-4^#^
		Mast cell	Sodium cromoglycate, Intravenous immunoglobulin

*CNS, central nervous system; NKCC1, Na^+^-K^+^-2Cl^−^ cotransporter-1; ZT-1a, a highly effective and selective SPAK inhibitor; HOE-642, a Na^+^-H^+^ exchanger inhibitor; Compound RU-1355, a Na^+^-H^+^ exchanger inhibitor; RB-222, anti-VEGF neutralizing antibody; SEA0400, a specific inhibitor of the Na^+^-Ca^2+^ exchanger; BBB, blood–brain barrier; VEGF, vascular endothelial growth factor; Anti-miR-1, anti-microRNA-1; Antagomir, inhibitor of miRNA-1*.

# *Note that VEGF can both promote cerebral edema and have neuroprotective effects. Aquaporin-4 has a protective effect against vasogenic edema*.

## Author Contributions

YZ and XL designed and conceptualized the review. YY drafted and revised the manuscript. YL revised the manuscript. RY and JL retrieved and screened the review. All authors contributed to the manuscript and approved the final version.

## Conflict of Interest

The authors declare that the research was conducted in the absence of any commercial or financial relationships that could be construed as a potential conflict of interest.

## Abbreviations

BBB: The blood–brain barrier
Sur1-Trpm4: The sulfonylurea receptor 1-immediate receptor potential melatonin 4
NKCC1: the Na^+^-K^+^-2Cl&#8211 cotransporter protein-1
AQP4: Aquaporin-4
AQP1: Aquaporin-1
KCC: Na^+^-K^+^-cotransporter
KCC2: Na^+^-K^+^-cotransporter 2
VEGF: Vascular endothelial growth factor
MMP: Free radical metalloprotease
MMP-9: Free radical metalloprotease-9
MCAO: A middle cerebral artery occlusion model
H2S: Hydrogen sulfide
SPAK: SPS1-related proline/alanine-rich kinase
ZT-1a: 5-chloro-N-[5-chloro-4-((4-chlorophenyl)(cyano) methyl)-2-methylphenyl]-2-hydroxybenzamide
HOE-642: The specific Na^+^-H^+^exchange inhibitor
Compound RU-1355: The new inhibitor of Na^+^-H^+^ exchanger
miRNAs: MicroRNAs
SEA0400: 2-[4-[(2,5-Difluorophenyl)methoxy]phenoxy]-5-ethoxyaniline.
